# Efficacy of tolvaptan for chronic heart failure: Study protocol for a systematic review of randomized controlled trial

**DOI:** 10.1097/MD.0000000000014540

**Published:** 2019-02-15

**Authors:** Wei-qin Gao, Xiang-dong Meng, Ze Sun

**Affiliations:** First Ward of Cardiology Department, First Affiliated Hospital of Jiamusi University, Jiamusi, China.

**Keywords:** chronic heart failure, efficacy, randomized controlled trial, safety, tolvaptan

## Abstract

**Background:**

The protocol of this study will be proposed for systematic evaluation of the efficacy and safety of tolvaptan in the treatment of chronic heart failure (CHF).

**Methods:**

We will retrieve the following electronic databases for randomized controlled trials assessing the efficacy of tolvaptan in patients with CHF: PubMed, Embase, Cochrane Central Register of Controlled Trials, Web of Science, Scopus, Chinese Biomedical Literature Database, China National Knowledge Infrastructure, VIP Information, and Wanfang Data. Each database will be retrieved from inception to February 1, 2019 without any limitations. The entire process of study selection, data extraction, and methodological quality evaluation will be conducted by 2 independent authors.

**Results:**

The protocol of this proposed study will compare the efficacy and safety of tolvaptan in the treatment of patients with CHF. The outcomes will include all-cause mortality, change in body weight, urine output, change in serum sodium; and incidence of all adverse events.

**Conclusion:**

The findings of this proposed study will summarize the current evidence of tolvaptan for CHF.

**Ethics and dissemination:**

All data used in this systematic review will be collected from the previous published trials. Thus, no research ethics approval is needed for this study. The findings of this study will be published at a peer-reviewed journal.

**PROSPERO registration number:**

PROSPERO CRD42019120818.

## 1 Introduction

Chronic heart failure (CHF) is one of the most serious cardiovascular diseases all over the world.^[1–4]^ It often causes a series of cardiac dysfunctions, such as ejection dysfunction, decreased cardiac output, and increased intracardiac pressure function.^[5–7]^ Most importantly, this condition also results in high mortality, with about 50% within 5 years.^[8]^ Epidemiological studies have reported that its prevalence is about 1 to 2% among general population.^[9,10]^ The huge cost of CHF treatment also brings the greatest burden for both families and society.^[11–13]^

Several previous trials have reported that tolvaptan can be used to treat heart failure effectively.^[14–22]^ Although one systematic review has assessed efficacy and safety of tolvaptan for the treatment of acute heart failure,^[23]^ no systematic review and meta-analysis has been conducted to specifically explore the efficacy and safety of tolvaptan for CHF based on many published clinical trials of CHF.^[24–28]^ Therefore, in this proposed protocol of systematic review, we will specifically investigate the efficacy and safety of tolvaptan for the treatment of CHF.

## 2 Methods

### 2.1 Inclusion criteria for study selection

#### 2.1.1 Study types

This proposed study will include randomized controlled trials (RCTs) that have assessed all types of tolvaptan for the treatment of patients with CHF. No restrictions of the location, time, and language of published papers will be applied. However, we will not consider non-clinical studies, case studies, non-RCTs, and quasi-RCTs.

#### 2.1.2 Participants

All participants must be clinically diagnosed as CHF, and will be considered without any limitations of race, gender, and age.

#### 2.1.3 Interventions

The experimental group must have been treated with tolvaptan alone. The control group must have been treated with other therapies, but not any types of tolvaptan.

#### 2.1.4 Outcomes

The primary outcome includes all-cause mortality. The secondary outcomes consist of change in body weight, urine output, change in serum sodium; and incidence of all adverse events.

### 2.2 Strategy of literature retrievals

The literature search will be mainly based on the electronic databases of PubMed, Embase, Cochrane Central Register of Controlled Trials (CENTRAL), Web of Science, Scopus, Chinese Biomedical Literature Database, China National Knowledge Infrastructure, VIP Information, and Wanfang Data from inception to February 1, 2019 without any limitations. Additionally, clinical registry websites, and reference lists of included trials will also be retrieved. The sample of detailed search strategy for CENTRAL has been built and showed in Table 1. Similar search strategy will also be applied to the other electronic databases.

**Table 1:**
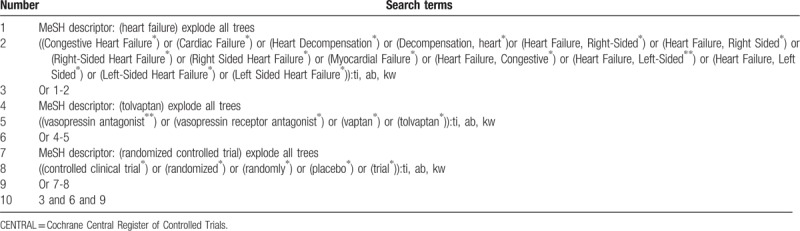
Search strategy applied in CENTRAL database.

### 2.3 Data extraction and methodological quality evaluation

#### 2.3.1 Study selection

**Figure 1:**
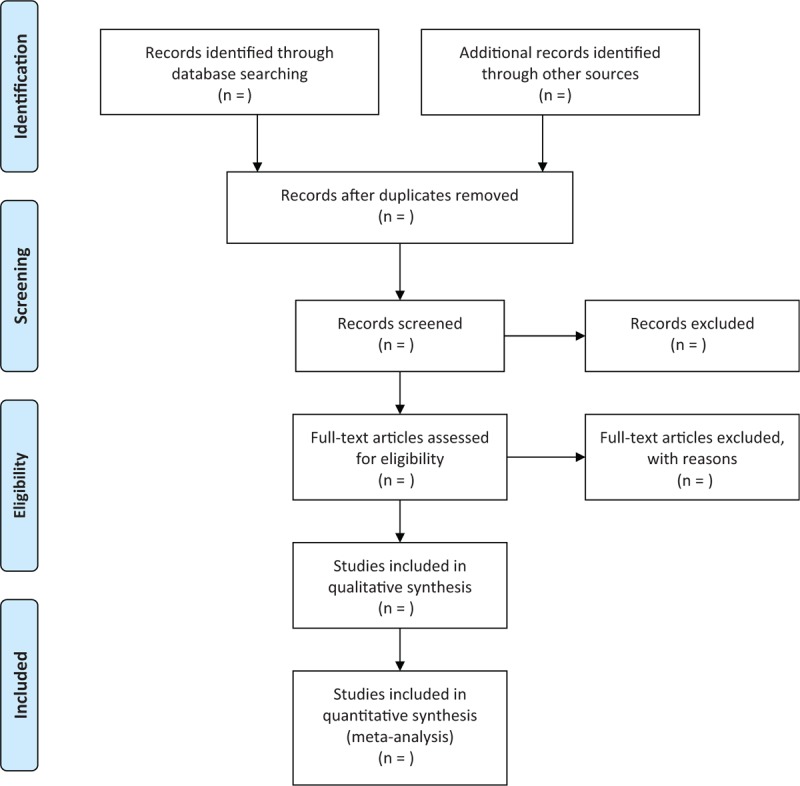
Flowchart of study selection.

Two independent authors will initially scan titles and abstracts of all potential studies. Then, full texts will be further read if there will be insufficient information to judge the study according to the inclusion criteria. Disagreements will be solved by discussion with other authors. The flowchart of study selection is demonstrated in Figure 1.

#### 2.3.2 Data extraction and management

Two independent authors will conduct data extraction according to the predefined standard data extraction form. The divergences will be resolved by consulting other authors. The form comprises of the following information: title, 1st author, year of publication, diagnosed criteria, inclusion and exclusion criteria, sample size, details of randomization, allocation, and blinding, intervention details, and outcomes. Any insufficient or missing data will be inquired by contacting the primary authors through email.

#### 2.3.3 Methodological quality evaluation

We will evaluate the methodological quality in each study by using Cochrane Risk of Bias Tool. Two independent authors will evaluate methodological quality for each study. Any divisions will be settled down by discussion with other authors.

### 2.4 Statistical analysis

We will use ReMan 5.3 software to pool and analyze the data. Continuous outcome data will be synthesized and presented as mean difference or standardized mean difference with 95% confidence intervals (CIs). Dichotomous outcome data will be synthesized and shown as risk ratio with 95% CIs.

Heterogeneity among included studies will be identified by *I*^*2*^ test. The *I*^*2*^ ≤50% is set as having reasonable heterogeneity, and a fixed-effect model will used to pool and analyze the data. The *I*^*2*^ >50% is considered as having significant heterogeneity, and a random-effect model will be utilized to pool and analyze the data. Then, subgroup analysis will be conducted to detect the possible reasons that may account for high heterogeneity. It will be carried out according to the different study characteristics, types of treatments, and outcome measurements. If it does not work, the pooled data and meta-analysis will not be performed. Instead, a narrative summary will be reported.

Additionally, sensitivity analysis will be carried out to identify the robustness of pooled results by removing low quality trials. Moreover, unit of analysis will be considered to conduct if cross-over studies included, and only 1st period of study data will be pooled and analyzed. Finally, reporting biases will also be performed by using funnel plot^[29]^ and Egg's regression,^[30]^ if more than 10 studies are included.

## 3 Discussion

The CHF is a very server cardiovascular disease, and often result in very high mortality. Tolvaptan is reported to treat heart failure effectively, especially for acute heart failure. However, no systematic review has addressed its efficacy and safety for the treatment of patients with CHF, although lots of high quality clinical trials have been published.^[24–28]^ Thus, this study will firstly and systematically explore the efficacy and safety of tolvaptan for CHF. It will provide the first rigorous summary evidence of tolvaptan for CHF across all published RCTs.

The data pooled results will provide a better understanding of efficacy and safety of tolvaptan for patients with CHF. Its findings will inform our understanding of the value of tolvaptan in treating CHF outcomes. Additionally, it may also provide helpful evidence for clinical practice and future studies.

## Acknowledgments

This study is supported by the Heilongjiang Provincial Health Department Scientific Research Project (2011-354). The funder had no role in the design, execution, or writing of the study.

## Author contributions

**Conceptualization:** Wei-qin Gao, Xiang-dong Meng, Ze Sun.

**Data curation:** Wei-qin Gao, Xiang-dong Meng, Ze Sun.

**Formal analysis:** Wei-qin Gao, Ze Sun.

**Funding acquisition:** Wei-qin Gao, Xiang-dong Meng.

**Investigation:** Xiang-dong Meng.

**Methodology:** Wei-qin Gao, Xiang-dong Meng, Ze Sun.

**Project administration:** Xiang-dong Meng.

**Resources:** Wei-qin Gao, Xiang-dong Meng, Ze Sun.

**Software:** Wei-qin Gao, Ze Sun.

**Supervision:** Xiang-dong Meng.

**Validation:** Wei-qin Gao, Xiang-dong Meng, Ze Sun.

**Visualization:** Wei-qin Gao, Xiang-dong Meng, Ze Sun.

**Writing – original draft:** Wei-qin Gao, Xiang-dong Meng.

**Writing – review & editing:** Wei-qin Gao, Xiang-dong Meng, Ze Sun.
